# Unilateral biportal endoscopic versus microscopic discectomy in degenerative lumbar spinal stenosis: A prospective cohort study

**DOI:** 10.1097/MD.0000000000042594

**Published:** 2025-05-23

**Authors:** Xinwen Feng, Bin Wang, Jiangping Ding, Ben Niu, Wumaier Muhetaer, Hongtao Yang, Rong Chen, Chao Ma

**Affiliations:** a Department of Spinal Surgery, Xinjiang Bazhou People’s Hospital, Korla, Xinjiang Uygur Autonomous Region, China.

**Keywords:** degenerative lumbar spinal stenosis, MED, microendoscopic discectomy, UBE, unilateral biportal endoscopic

## Abstract

**Background:**

Unilateral biportal endoscopic discectomy (UBE) and microendoscopic discectomy (MED) are well-established minimally invasive techniques for managing single-segment degenerative lumbar spinal stenosis (DLSS). However, current evidence from evidence-based medicine remains insufficient to conclusively demonstrate the comparative advantages of these methods.

**Methods:**

A total of 145 patients diagnosed with single-segment DLSS were enrolled from the spinal surgery department of Xinjiang Bazhou People’s Hospital between January 2022 and August 2024. Fourteen patients were lost to follow-up, leaving 131 valid cases. Participants were divided into 2 groups: UBE (n = 70) and MED (n = 61), based on the surgical approach. The study compared the demographic and clinical characteristics of both groups (gender, age, disease duration, height, weight, BMI, and affected segment), perioperative metrics (operation time, blood loss, and hospital stay), and clinical outcomes at various time points (preoperatively, 3 days postoperatively, 1-month postoperatively, 3 months postoperatively, 1-year postoperatively, and 2 years postoperatively), including visual analogue scale (VAS) scores for back and leg pain, Oswestry disability index (ODI) scores, and imaging parameters (preoperative and postoperative disc height and dural sac expansion area).

**Results:**

All patients completed follow-up for over 2 years. The UBE group demonstrated significantly shorter operation times, reduced blood loss, and shorter hospital stays compared to the MED group (*P* < .05). No significant differences in VAS scores for back and leg pain or ODI scores were found between the groups at preoperative and postoperative time points (*P* > .05). Both groups showed significant improvements in VAS scores for back and leg pain and ODI scores at all postoperative time points relative to preoperative levels (*P* < .05). No significant differences in dural sac expansion area or disc height (preoperative and postoperative) were noted between the groups (*P* > .05).

**Conclusion:**

Both UBE and MED are effective treatments for single-segment DLSS, achieving substantial spinal canal decompression and improvement in clinical symptoms. UBE, however, offers advantages over MED in terms of shorter surgical time, reduced blood loss, and shorter hospital stays.

## 1. Introduction

Degenerative lumbar spinal stenosis (DLSS) is a clinical syndrome characterized by narrowing of the lumbar spinal canal due to factors such as lumbar disc herniation, ligamentum flavum hypertrophy, and facet joint proliferation, leading to compression of the spinal cord or nerve roots. The primary symptoms include lower limb pain, numbness, and intermittent claudication.^[1]^ With the shift in lifestyle and the increasing aging population, DLSS has become a significant condition impacting the quality of life in middle-aged and elderly individuals.

Posterior lumbar decompression remains the standard surgical approach for DLSS, increasing the spinal canal volume through resection of the lamina, ligamentum flavum, intervertebral disc, and other structures, resulting in effective decompression.^[2]^ However, the destruction of the posterior spinal column required to achieve sufficient decompression raises concerns regarding postoperative spinal stability, a critical issue that warrants attention.^[3]^ Additionally, the high cost and complexity of the procedure limit its widespread adoption and application.

With advancements in minimally invasive surgery and improvements in spinal endoscopic technology, spinal endoscopic surgery has gained widespread acceptance in recent years. Initially used for disc herniation, its scope has now extended to treating spinal stenosis and spinal instability. Unilateral biportal endoscopic discectomy (UBE) and microendoscopic discectomy (MED) are 2 prominent techniques for applying spinal endoscopy to treat DLSS.^[4,5]^ MED, having been performed in China for several years, has evolved from a procedure focused solely on disc resection to one capable of spinal canal decompression. UBE, though a more recent development, has been increasingly adopted in major hospitals.

Both techniques are viable options for treating single-segment DLSS. However, there remains insufficient evidence-based data to definitively compare their relative advantages and limitations. Consequently, our center has initiated a prospective cohort study to assess the clinical outcomes of UBE-assisted and microendoscopic-assisted treatments for single-segment DLSS.

## 2. Methods

### 2.1. Trial design

This single-center, prospective cohort study was conducted at Xinjiang Bazhou People’s Hospital from January 2022 to August 2024. All patients were enrolled and underwent corresponding surgical procedures in August 2022. Approval was obtained from the Ethics Committee of Xinjiang Bazhou People’s Hospital (No.: BZRMYY(2024)52) and conducted in accordance with the Declaration of Helsinki. All participants and their families were thoroughly briefed on the study procedures and provided written informed consent.

### 2.2. Inclusion criteria

Patients are eligible for study inclusion if they

(1) CT or MRI confirms single-segment lumbar spinal stenosis with intermittent claudication and nerve root signs/symptoms corresponding to the stenotic level.
(2) Failure of standard nonsurgical treatment (>6 weeks).
(3) Neurogenic intermittent claudication or radicular symptoms in the lower extremities affecting activities of daily living.
(4) Ability to comply with regular follow-up assessments for over 2 years post-surgery.

### 2.3. Exclusion criteria

Patients were excluded if they

(1) Presence of cauda equina syndrome or progressive neurological deficits requiring urgent surgery.
(2) Concurrent spinal pathologies requiring surgical intervention other than DLSS, such as spinal tumors, severe spinal deformities, or spondylolisthesis (≥grade II).
(3) History of other prior spinal surgeries.
(4) Pregnancy.
(5) Systemic comorbidities compromising surgical outcomes or perioperative safety (e.g., coronary artery disease, respiratory dysfunction).

### 2.4. Interventions

Patients meeting the inclusion criteria were assigned to either the UBE group or the MED group based on the surgical approach. The surgical procedure for the UBE group was as follows: Under general anesthesia, the patient was positioned prone. The C-arm was used to identify the responsible interspace and the inner borders of the upper and lower pedicles. Transverse incisions, approximately 2 to 3 cm in length, were made at the intersection points of the projection lines of the pedicle inner edges, 1.5 cm above and 2 cm below the intervertebral space horizontal line. Sequential incisions were made through the skin, subcutaneous tissue, and lumbar dorsal fascia. A dilatation cannula was inserted to establish both the observation and working channels, followed by the insertion of an endoscope and plasma radiofrequency electrotome. The articular processes and portions of the upper and lower laminae were resected, the ligamentum flavum excised, the dura mater exposed, and the nerve roots decompressed. The nucleus pulposus was removed. In cases of bilateral symptoms, decompression was performed bilaterally using the same technique. The MED group underwent the following procedure: After general anesthesia, the patient was placed in the prone position, and the C-arm was used to locate the intervertebral space and pedicles. A 1.5 to 2 cm incision was made 1.5 to 2 cm lateral to the midline of the targeted intervertebral space. Subsequent incisions were made through the skin, subcutaneous tissue, and lumbar dorsal fascia. Sequential dilators were used to expand the space, and a channel was established. Resection was performed on portions of the lower edges of the upper and lower laminae and the medial edges of the articular processes at the targeted intervertebral space. The ligamentum flavum was excised, the dura mater exposed, and the nucleus pulposus removed. All surgical procedures were performed by the same experienced surgical team. Postoperatively, patients received routine anti-inflammatory, analgesic, nerve-nourishing, and basic disease management treatments, along with guidance for functional rehabilitation. Clinical data were collected at baseline, 3 days post-surgery, 1-month postoperatively, 3 months post-surgery, 1-year post-surgery, and 2 years post-surgery for evaluation.

### 2.5. Variables and assessments

(1) Perioperative indicators: Operation time, blood loss, and hospital stay.
(2) Clinical efficacy: The Visual Analogue Scale (VAS) was used to assess low back pain and leg pain at preoperative, 3-day, 1-month, 3-month, 1-year, and 2-year follow-up points. The VAS, a 10-cm ruler, allowed patients to rate their subjective pain, with higher scores indicating more severe pain. The Oswestry Disability Index (ODI) evaluated lumbar function improvement at the same time points. The ODI comprises 10 items, each rated from 0 to 5 points. The total score was calculated, with a lower ratio indicating better function.
(3) Imaging indicators: Lumbar MRI was performed preoperatively and 3 days postoperatively to evaluate the dural sac area. The area was defined by the dural sac boundary on T2W1 axial images, ensuring both measurements were on the same plane.^[6]^ The dural sac expansion was calculated as the postoperative area minus the preoperative area. Lumbar anteroposterior and lateral X-rays were taken preoperatively and 3 days postoperatively to measure intervertebral disc height changes. All imaging parameters were measured by 2 professional radiologists using the hospital’s imaging system. In case of significant measurement discrepancies, a third radiologist resolved the disagreement, and the average value was used for analysis.

### 2.6. Statistical analysis

The obtained data were statistically evaluated with SPSS Version 25.0 program (SPSS, Chicago). The level of significance was accepted as *P < *.05. Quantitative data were expressed in the form of mean ± standard deviation ($\bar{X}\pm SD$). All data were tested for normality (Shapiro–Wilk test). The data were not suitable for normal distribution. The chi-square test was used for comparisons of demographic information. The Mann–Whitney *U* test was used to determine the differences between the groups, and Kruskal–Wallis test was used to compare the data measured at different times. The post hoc Dunn test was applied to intra group analysis, aiming to prevent type-1 errors.

## 3. Results

**Figure 1.**
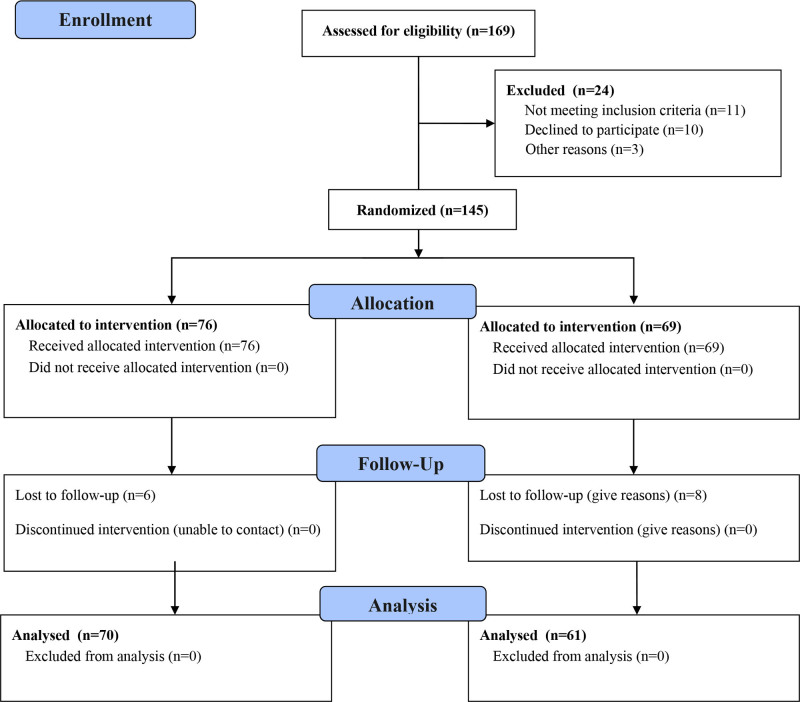
Flowchart of the trial design.

A total of 145 patients with single-segment DLSS were initially included in the study. During the follow-up period, 14 patients were lost to follow-up, leaving 131 valid cases for analysis. Based on the surgical approach, the patients were assigned to either the UBE group (n = 70) or the MED group (n = 61). In the UBE group, there were 37 male and 33 female patients, with an average age of 63.44 ± 6.65 years and a disease duration of 23.56 ± 8.57 months. No statistically significant differences were observed in height, weight, or BMI between the 2 groups. The responsible segment distribution included 25 cases at L_3/4_, 22 at L_4/5_, and 23 at L_5_/S_1_. In the MED group, 32 male and 29 female patients were included, with an average age of 62.39 ± 9.11 years and a disease duration of 23.67 ± 4.80 months. The responsible segment distribution in this group was 19 cases at L_3/4_, 24 at L_4/5_, and 18 at L_5_/S_1_. Both groups had a follow-up period exceeding 2 years. Patient demographics for both groups are detailed in Table 1 and Figure 1.

**Table 1:** Comparison of general data between the 2 patient groups.

Demographics	UBE (n = 70) ($\bar{X}\pm SD$)	MED (n = 61) ($\bar{X}\pm SD$)	*P*-value*
Age (yr)	63.44 ± 6.65	62.39 ± 9.11	.45
Height (m)	1.65 ± 0.10	1.65 ± 0.09	.97
Weight (kg)	69.47 ± 8.86	69.32 ± 8.37	.84
BMI (kg/m^2^)	25.33 ± 0.43	25.24 ± 0.42	.23
Duration (mo)	23.56 ± 8.57	23.67 ± 4.80	.74

	n	n	*P*-value†
Gender			
Male	37	32	.96
Female	33	29	
Lesional segment			
L_3/4_	25	19	.64
L_4/5_	22	24	
L_5_/S_1_	23	18	

BMI = body mass index, MED = microendoscopic discectomy, ODI = oswestry disability index, UBE = unilateral biportal endoscopic discectomy, VAS = visual analogue scale.

* Mann–Whitney *U* test.

† Chi-square test.

### 3.1. Perioperative indicators

The average operation time was 57.79 ± 5.62 minutes in the UBE group and 66.74 ± 8.35 minutes in the MED group. Intraoperative blood loss was 50.36 ± 8.39 mL in the UBE group and 97.89 ± 12.77 mL in the MED group, with a statistically significant difference (*P* < .001). The hospital stay was 5.77 ± 0.78 days in the UBE group and 6.80 ± 1.30 days in the MED group, with a significant difference observed (*P* < .05). These results are presented in Table 2.

**Table 2:** Comparison of perioperative indicators between the 2 patient groups.

Parameters	UBE (n = 70) ($\bar{X}\pm SD$)	MED (n = 61) ($\bar{X}\pm SD$)	*P*-value*
Operation time (min)	57.79 ± 5.62	66.74 ± 8.35	<.001
Blood loss (mL)	50.36 ± 8.39	97.89 ± 12.77	<.001
Hospital stay (d)	5.77 ± 0.78	6.80 ± 1.30	<.001

MED = microendoscopic discectomy, UBE = unilateral biportal endoscopic discectomy.

* Mann–Whitney *U* test.

### 3.2. VAS and ODI

No statistically significant differences were observed in the VAS scores for low back pain, VAS scores for leg pain, or ODI scores between the 2 groups at preoperative, 3-day, 1-month, 3-month, 1-year, and 2-year follow-up time points (*P* > .05). These findings are presented in Table 3.

**Table 3:** Comparison of low back pain VAS scores, leg pain VAS scores, and ODI scores between the 2 patient groups.

Parameters	UBE (n = 70) ($\bar{X}\pm SD$)	MED (n = 61) ($\bar{X}\pm SD$)	*P* value*
VAS of back pain ($\bar{X}\pm SD$, cm)			
Preoperatively	7.44 ± 1.09	7.48 ± 1.18	.87
3 d postoperatively	2.39 ± 0.69	2.33 ± 0.68	.63
1 mo postoperatively	1.59 ± 0.55	1.57 ± 0.50	.90
3 mo postoperatively	1.49 ± 0.56	1.57 ± 0.50	.35
1 yr postoperatively	1.49 ± 0.61	1.54 ± 0.50	.57
2 yr postoperatively	1.40 ± 0.55	1.40 ± 0.49	.94
*P*-value†	<.001	<.001	
VAS of leg pain ($\bar{X}\pm SD$, cm)			
Preoperatively	7.44 ± 1.09	7.48 ± 1.18	.87
3 d postoperatively	3.91 ± 0.88	3.89 ± 0.80	.84
1 mo postoperatively	2.47 ± 0.50	2.52 ± 0.50	.55
3 mo postoperatively	1.73 ± 0.78	1.95 ± 0.80	.11
1 yr postoperatively	1.50 ± 0.50	1.52 ± 0.50	.78
2 yr postoperatively	1.49 ± 0.50	1.59 ± 0.49	.24
*P* value†	<.001	<.001	
ODI ($\bar{X}\pm SD$, %)			
Preoperatively	69.17 ± 5.09	70.44 ± 6.15	.20
3 d postoperatively	46.96 ± 4.78	46.39 ± 4.62	.50
1 mo postoperatively	15.00 ± 2.73	15.51 ± 2.88	.30
3 mo postoperatively	13.93 ± 1.92	14.13 ± 2.42	.59
1 yr postoperatively	13.06 ± 1.30	13.41 ± 2.00	.23
2 yr postoperatively	11.93 ± 1.44	12.23 ± 1.33	.22
*P*-value†	<.001	<.001	

MED = microendoscopic discectomy, ODI = oswestry disability index, UBE = unilateral biportal endoscopic discectomy, VAS = visual analogue scale.

* Mann–Whitney *U* test.

† Dunn test.

### 3.3. Imaging indicators

**Figure 2.**
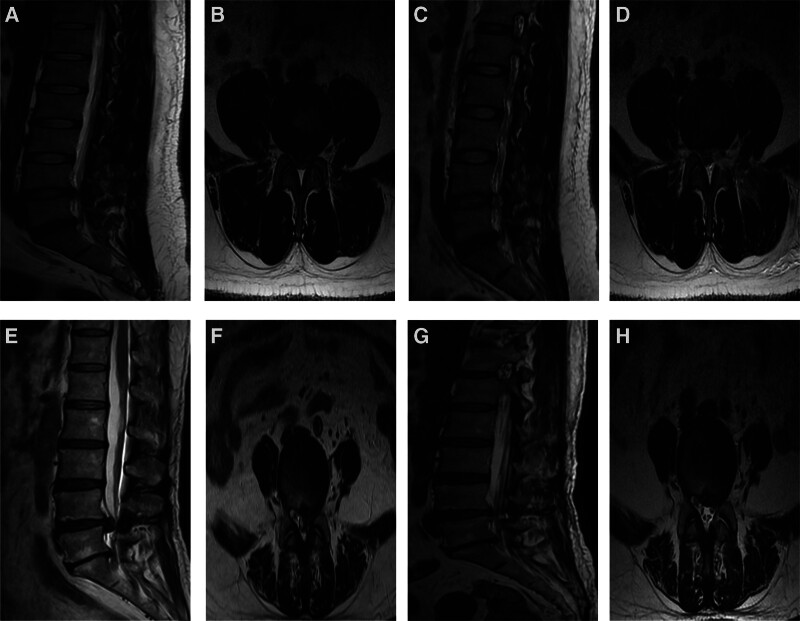
Typical cases. (A–D) Preoperative (A and B) and postoperative MRI (C and D) of UBE group patients; (E–H) preoperative (E and F) and postoperative MRI (G and H) of MED group patients.

The average expansion area of the dural sac was 45.52 ± 3.82 mm^2^ in the UBE group and 46.19 ± 3.52 mm^2^ in the MED group. The preoperative intervertebral disc height was 11.35 ± 0.57 mm in the UBE group and 11.29 ± 0.63 mm in the MED group. Postoperatively, the intervertebral disc height was 10.47 ± 0.74 mm in the UBE group and 10.27 ± 0.27 mm in the MED group. No statistically significant differences were found in the average dural sac expansion area, nor in the preoperative and postoperative intervertebral disc heights between the 2 groups (*P* > .05). These results are presented in Table 4, with typical cases illustrated in Figure 2.

**Table 4:** Comparison of imaging indicators between the 2 patient groups.

	UBE (n = 70) ($\bar{X}\pm SD$)	MED (n = 61) ($\bar{X}\pm SD$)	*P*-value*
Dural sac expansion area (mm^2^)	45.52 ± 3.82	46.19 ± 3.52	.11
Disc height (mm)			
Preoperatively	11.35 ± 0.57	11.29 ± 0.63	.50
Postoperatively	10.47 ± 0.74	10.27 ± 0.27	.83
*P*-value*	<.001	<.001	

* Mann–Whitney *U* test.

## 4. Discussion

Foley and Smith first introduced the microendoscopic system for treating lumbar disc herniation in 1997,^[7]^ subsequently reporting the clinical outcomes of 100 patients treated with MED the following year.^[8]^ MED utilizes an interlaminar approach to enlarge the nerve root canal, effectively relieving nerve root compression and removing the degenerated nucleus pulposus while minimizing damage to surrounding bony structures and soft tissues.^[9,10]^ Compared to traditional open surgeries, MED offers advantages such as higher safety, reduced trauma, shorter operation time, and faster postoperative recovery. Over nearly 3 decades of development, MED has become a widely accepted method for treating lumbar disc herniation, DLSS, and similar conditions, significantly advancing the minimally invasive approach in spinal surgery. The technique works by sequentially placing dilators to expose the optimal surgical window, followed by the insertion of a tubular retractor and endoscope. The magnification provided by the endoscope allows for clear visualization of the surgical area, enabling decompression and disc removal as required. However, the single-channel nature of MED limits the operating space and provides a relatively narrow surgical field. This requires a high level of spatial awareness from surgeons, especially since the 2-dimensional view may prove challenging. Additionally, in cases requiring bilateral decompression, the operating table needs to be tilted to enhance the field of vision for the opposite side, making the learning curve for young surgeons relatively steep.

De Antoni first documented a surgical method resembling modern UBE in 1996,^[11]^ though it did not gain significant attention at the time. In recent years, with advancements in spinal endoscopy technology, UBE has been progressively optimized and integrated into spinal surgery. The UBE technique combines elements of both open spinal surgery and spinal endoscopy. It offers a broad range of indications similar to open surgery while incorporating the minimally invasive and precise advantages of spinal endoscopy, which has garnered significant clinical interest.^[12]^ The UBE approach employs a dual-channel system: the observation and working channels. In comparison to MED, UBE allows surgeons to operate more freely with fewer restrictions, facilitating direct manipulation of instruments. This dual-channel feature provides notable benefits in procedures such as intervertebral disc nucleus pulposus removal, spinal canal decompression, and intervertebral fusion.^[13,14]^ Additionally, the dual-channel system enhances the visualization of the surgical area. The 30° endoscope, with its small diameter, high sensitivity, and large operating range, offers a broader operating space, enabling safe and efficient bilateral nerve root decompression. This reduces the complexity and duration of surgery for complex DLSS cases and minimizes intraoperative bleeding. A total of 131 valid cases were included in this study, comprising 70 patients in the UBE group and 61 patients in the MED group. The baseline characteristics of both groups were comparable. Comparison of intraoperative blood loss, operation time, and hospital stay revealed that the UBE group outperformed the MED group, consistent with previous studies.^[15,16]^ Some researchers have argued that UBE requires more time than MED.^[17]^ However, this study did not observe such a difference, which may be attributed to the necessity of bilateral decompression in many cases. The operational characteristics of MED contributed to the longer procedure time. Additionally, the surgeons’ level of proficiency likely played a role.

In evaluating clinical effectiveness, the VAS and ODI scores showed significant improvement for both low back and leg pain in both groups postoperatively, with no significant difference between the UBE and MED groups. Similarly, when comparing the expansion area of the dural sac and the height of the intervertebral disc, no significant differences were found between the 2 groups. The dural sac area was measured through lumbar MRI, which provides an objective measure of spinal canal decompression and is widely used for postsurgical imaging evaluation.^[18]^ These findings suggest that both surgical techniques offer comparable decompression outcomes. A prospective study demonstrated that UBE significantly increases the dural sac area, achieving effective decompression,^[19]^ a result similarly confirmed for MED in other studies.^[20]^ Further research has investigated the clinical efficacy of UBE and MED for single-segment DLSS. A prospective randomized controlled trial following 64 patients who underwent either UBE or MED for a year found no significant differences in postoperative clinical outcomes between the 2 techniques.^[21]^ However, other studies have reached differing conclusions. For example, a study involving 154 patients with single-segment DLSS, who were assigned to UBE or MED, found that the UBE group achieved lower ODI scores at multiple postoperative time points following a 2-year follow-up.^[22]^ A meta-analysis encompassing 9 relevant studies compared the efficacy of UBE and MED, revealing that UBE was more effective in reducing the VAS score for low back pain. However, no significant difference was observed between the 2 groups in terms of ODI scores.^[23]^ Both surgical methods are effective in achieving spinal canal decompression, as supported by imaging indicators, which provide objective evidence of the decompression outcomes and serve as the primary means of evaluating the advantages and disadvantages of each method. However, VAS and ODI scores, which are subjective, may introduce bias into the conclusions. Additionally, the lower VAS scores for low back pain observed in the UBE group may be attributed to more extensive damage to the paraspinal soft tissues during MED procedures.

Dural tear is the most common complication for both UBE and MED, and it can even lead to pseudomeningocele. This complication is often linked to the use of surgical tools (such as scraping by a curette), the application of tools like plasma radiofrequency during surgery, and the experience level of the surgeon.^[24]^ Epidural hematoma is also a relatively frequent complication, with 1 study reporting an incidence of 8.4% following UBE, which is higher than in traditional open spinal surgeries.^[25]^ Other complications associated with spinal endoscopic procedures include recurrence of intervertebral disc herniation.^[26]^ In the present study, none of these complications were observed, which was attributed to careful case selection before surgery and meticulous execution during the procedures.

In conclusion, both UBE and MED are effective treatments for single-segment DLSS, demonstrating the ability to achieve substantial spinal canal decompression and alleviate clinical symptoms. However, UBE offers advantages over MED, including shorter operation time, reduced blood loss, and a shorter hospital stay. This study has some limitations, including a relatively small sample size, which may introduce bias in the final conclusions. Moreover, the difficulty of achieving effective randomization in single-center studies is a challenge in surgical research. Future studies should involve higher-quality, multi-center, prospective randomized controlled trials to provide more robust evidence.

## Author contributions

**Data curation:** Xinwen Feng, Bin Wang, Chao Ma.

**Investigation:** Bin Wang, Jiangping Ding, Ben Niu, Muhetaer Wumaier, Hongtao Yang, Rong Chen, Chao Ma.

**Methodology:** Xinwen Feng, Bin Wang, Jiangping Ding, Ben Niu, Muhetaer Wumaier, Hongtao Yang, Rong Chen, Chao Ma.

**Software:** Bin Wang, Ben Niu, Rong Chen.

**Supervision:** Xinwen Feng, Bin Wang, Ben Niu, Hongtao Yang, Rong Chen, Chao Ma.

**Validation:** Muhetaer Wumaier, Rong Chen.

**Visualization:** Muhetaer Wumaier, Hongtao Yang, Rong Chen, Chao Ma.

**Writing – original draft:** Xinwen Feng, Chao Ma.

**Writing – review & editing:** Xinwen Feng, Chao Ma.
